# Synthesis and Drug Delivery Application of Thermo- and pH-Sensitive Hydrogels: Poly(β-CD-*co*-*N*-Isopropylacrylamide-*co*-IAM)

**DOI:** 10.3390/ma9121003

**Published:** 2016-12-11

**Authors:** Syang-Peng Rwei, Tuan Huynh Nguyen Anh, Whe-Yi Chiang, Tun-Fun Way, Yung-Jia Hsu

**Affiliations:** Institute of Organic and Polymeric Materials, National Taipei University of Technology, Taipei 10608, Taiwan; tuanhna@hcmute.edu.vn (T.H.N.A.); wheyichiang@gmail.com (W.-Y.C.); tfway@itri.org.tw (T.-F.W.); arvinhsu1992@gmail.com (Y.-J. H.)

**Keywords:** thermo- and pH-sensitive, β-cyclodextrin, *N*-isopropylacrylamide, intelligent materials, hydrogel, LCST, drug release, atorvastatin

## Abstract

Copolymerization of *N*-isopropylacrylamide (NIPAM), itaconamic acid (IAM; 4-amino-2-methylene-4-oxobutanoic acid) and β-cyclodextrin was investigated in this study. β-cyclodextrin was at first modified by reacting with allyl glycidyl ether to substitute its OH end groups with moieties containing double bonds to facilitate the subsequent radical copolymerization with NIPAM and IAM. It was reported that poly(NIPAM-IAM) can respond to the change of temperature as well as pH value. In this study, the structure of β-cyclodextrin was introduced to poly(NIPAM-IAM) copolymers because of its cavity structure capable of encapsulating a variety of drug molecules. The tri-component copolymers, poly(CD-NIPAM-IAM), were synthesized with different monomeric ratios of NIPAM/IAM/β-CD and the hydrogels of the tri-component copolymers were also synthesized by additionally adding *N*,*N*′-methylenebisacrylamide as a cross-linking agent. The results show that the lower critical solution temperature (LCST) of the copolymer (or hydrogel) increases as the molar fraction of IAM increases. The transmission electron microscopic (TEM) images of linear copolymers (no cross-linking) show that molecules undergo self-assembly to have a distinct core–shell structure, compared to poly(CD-NIPAM) which contains no IAM. On the other hand, the scanning electron microscopic (SEM) images of hydrogels show that the pores gradually become sheet-like structures as the molar fraction of IAM increases to enhance the water absorption capacity. In order to exhibit the thermal and pH sensitivities of poly(CD-NIPAM-IAM) as the drug carrier, the drug release of the newly synthesized hydrogels at 37 °C and different pH values, pH = 2 and pH = 7.4, was investigated using atorvastatin which was used primarily as a lipid-lowering drug. The drug release experimental result shows that poly(CD-NIPAM-IAM) as a drug carrier was pH-sensitive and has the largest release rate at pH = 7.4 at 37 °C, indicating it is useful to release drugs in a neutral or alkaline (intestinal) environment.

## 1. Introduction

A smart material is responsive to a physical or chemical act, such as light, heat, electric force, magnetic force, acid–base and so forth [1,2,3] and has the potential in biomedical applications such as drug release [4,5,6,7,8,9]. The drug release mechanism [10,11] usually includes chemical-responsive (for example glucose or antigen responsive [12]), light-responsive [13,14], electric-responsive [15], magnetic responsive [8,9], temperature-responsive or pH-responsive systems. Especially, the temperature responsive polymers, such as poly(*N*-isopropylacrylamide) (poly(NIPAM)) families [16,17,18,19,20,21,22], have a lower critical solution temperature (LCST) close to the human body temperature to be extensively studied in drug release applications. Poly(NIPAM) is widely used as a thermo- responsive carrier to control drug delivery but a carrier having not only thermo-sensitivity but also pH sensitivity is even more favorable for controlled drug release, particularly for the application of site-specific drug release. Therefore, *N*-isopropylacrylamide (NIPAM) is often used as a monomer to contribute the thermo-sensitive or thermo-responsive composition units to a multiple responsive copolymer, specifically thermo- and pH-sensitive copolymer [23,24,25,26]. A thermo- and pH-dual stimuli-responsive polymer can respond to not only the temperature change but also the pH change of the environment by varying its solubility, dimension, etc. to carry or release drugs. 

By copolymerization of two or more monomers containing two or more different functional groups, the resulting copolymer can be expected to have more functionalities as a smart polymer or environmental responsive polymer. In previous studies, itaconamic acid (IAM; 4-amino-2-methylene-4-oxobutanoic acid) was used to contribute pH-sensitivity to *N*-isopropylacrylamide-based (NIPAM-based) copolymers [23,24,25]. The applicability of controlled drug delivery of copolymers having various structures, such as hyperbranched or star structures, was investigated. A hydrogels is formed by crosslinking polymer chains to have a three-dimensional network and has the hydrophilic structure so that it is capable of holding a large amount of water and also could protect the drug from hostile environments. The poly(CD-NIPAM-IAM) hydrogels were first synthesized and studied in this work. Hydrogels, as a drug carrier, have been limited to carry hydrophilic drugs rather than hydrophobic drugs due to the limited quantity/homogeneity of loaded hydrophobic drugs in hydrogel matrices. To improve drug loading capacity, various strategies, such as formation of micellar structures (hydrophobic core–hydrophilic shell), copolymerization with monomers contributing hydrophobic or lipophilic composition units, can be adopted.

On the other hand, cyclodextrin made up of sugar molecules bound together in a ring has been used as a drug carrier because it is nontoxic and not only has a hydrophobic cavity and a hydrophilic outer shell but also has many hydroxyl groups on its outer shell. As a drug carrier, the hydrophobic cavity of cyclodextrin can enhance the solubility for poorly water soluble drugs by hiding the hydrophobic functionalities in the interior cavity. Therefore, cyclodextrin and its derivatives have been designed to exhibit various characteristics including stimuli-responsiveness and drug release [27,28]. To provide more functionality of cyclodextrin as a drug carrier, this work used NIPAM, IAM [29] and cyclodextrin as monomers to give thermal and pH sensitivities as well as the cavity structure.

The copolymers in the current work were synthesized using NIPAM as a monomer to give the thermo-sensitive or thermo-responsive composition units, using IAM [29] as a monomer to give the pH-sensitive composition units and using β-cyclodextrin as a monomer to give composition units containing the hydrophobic cavity to carry poorly water soluble drugs. The properties of poly(CD-NIPAM-IAM) were studied and the results show the possibility of biomedical applications.

## 2. Experimental

### 2.1. Materials

*N*-isopropylacrylamide (NIPAM; 97%; C_6_H_11_NO) obtained from Sigma-Aldrich Inc. (St. Louis, MI, USA) was purified through recrystallization from n-hexane twice. Itaconamic acid was prepared according to the method disclosed in US patent publication No. 2013/0172490 [29]. β-Cyclodextrin (purity 99%) was obtained from TCI Co. (Tokyo, Japan) 2,2′-Azobis(isobutyronitrile) (AIBN) (purity 98%) was obtained from Aldrich Chemical Corp. without further purification. *N*,*N*′-Methylenebisacrylamide (MBA) and allyl glycidyl ether (AGE) were obtained from Alfa Aesar Co. (Tewksbury, MA, USA) Ammonium persulfate (APS) was obtained from Aencore Chemical PTY. Ltd. (Surrey Hills, Australia) and *N*,*N*,*N*′,*N*′-tetramethyl-ethane-1,2-diamine (TEMED) was obtained from Acros Organics Co. (Newark, NJ, USA) Tetrahydrofuran (THF) and dimethyl formamide (DMF) were obtained from ECHO Co. (Taipei, Taiwan) and dimethyl sulfoxide (DMSO) was obtained from TEDIA Co. (distributed by Echo Co., Taipei, Taiwan) Toluene was obtained from J.T. Baker Co. (Radnor, PA, USA) The buffer solution of pH = 4, obtained from Aldrich, was prepared from potassium hydrogen phthalate; the buffer solution of pH = 7, obtained from Aldrich, was prepared from potassium dihydrogen phosphate and disodium hydrogen phosphate; and the buffer solution of pH = 12, obtained from Aldrich, was prepared from di-sodium hydrogen phosphate and sodium hydroxide solution. The buffer solution of pH 7.4, obtained from Aldrich, was prepared from phosphate buffered saline.

### 2.2. Synthesis of Allyloxy-CD

**Figure 1:**
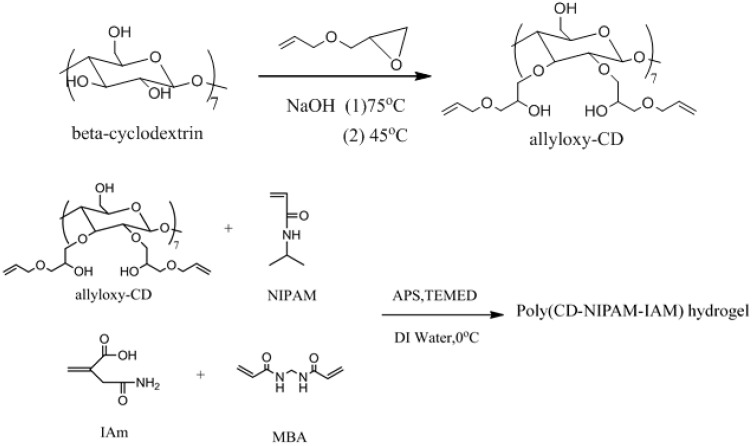
Synthesis of heptakis [2,3-di-*O*-(3-allyloxy-2-hydroxypropyl)]-β-CD (allyloxy-β-CD) and poly(CD-NIPAM-IAM).

Figure 1 shows the synthesis scheme of heptakis [2,3-di-*O*-(3-allyloxy-2-hydroxypropyl)]-β-CD (allyloxy-CD). In a reaction flask, a 20 wt % NaOH solution containing 10.5 g of NaOH and β-cyclodextrin (β-CD) (2.838 g, 2.5 mmol) were added. A reflux tube was attached to the reaction flask and the reaction flask was cooled in a 5 °C water bath. At 75 °C, the reaction flask was purged with nitrogen gas and stirred at the same time to completely be dissolved until having no viscous solid residue and then was cooled to 45 °C. At 45 °C, allyl glycidyl ether (AGE) (8.988 g, 78.7 mmol) was gradually added drop by drop and the mixture was stirred for 12 h until the reaction was complete. After the reaction was complete, 50 mL of ethanol was added and hydrochloric acid was added to neutralize the reaction solution. A rotary evaporator was used to remove water and ethanol. Fifty milliliters of Ethanol was added to re-dissolve the residue of the reaction solution after evaporation and vacuum-filtration were used to remove the by-products. Finally, ethanol in the solution was removed by the rotary evaporator and a yellow viscous liquid (allyloxy-CD) was obtained.

### 2.3. Synthesis of Poly(CD-NIPAM) and Poly(CD-NIPAM-IAM) Copolymers (CD-0, CD-8, CD-10 and CD-12)

Poly(CD-NIPAM) and poly(CD-NIPAM-IAM) copolymers were synthesized by radical polymerization. NIPAM and allyloxy-CD monomers with a molar ratio of 100/1 were polymerized. NIPAM (2.716 g, 24 mmol), allyloxy-CD (0.656 g, 0.24 mmol) (with a molar ratio of allyloxy-CD/NIPAM = 1/100), IAM (with a molar ratio of NIPAM/IAM = 100/0, 100/8, 100/10, or 100/12) and AIBN (0.0218 g, 0.1332 mmol) were dissolved in DMSO/toluene (30 mL) mixture in a 250 mL flask. Before polymerization, the flask was vacuumed and nitrogen-purged repeatedly. Then, the flask was kept at about 70 °C in the nitrogen environment for polymerization for 24 h. After the reaction was complete, the reaction mixture was added dropwise to diethyl ether for purification through precipitation. After filtration, light-yellow solids were obtained and vacuum-dried named as samples CD-0, CD-8, CD-10 and CD-12 for allyloxy-CD/IAM = 1/0, 1/8, 1/10 and 1/12, respectively.

### 2.4. Synthesis of Poly(CD-NIPAM) and Poly(CD-NIPAM-IAM) Hydrogels

NIPAM (1.358 g, 12 mmol), allyloxy-CD (0.328 g, 0.12 mmol) (with a molar ratio of allyloxy-CD/NIPAM = 1/100), IAM (with a molar ratio of NIPAM/IAM = 100/0, 100/1.67, 100/2.5, or 100/5) and MBA (0.0679 g, 0.44 mmol) were dissolved in 12.5 mL of deionized water (DI water) in a 250 mL flask. Before polymerization, the flask was vacuumed and nitrogen-purged for 20 min and placed in a 5 °C water bath. Then, 2.5 mL of APS (ammonium persulfate) solution (84 mM) and 5 mL of TEMED (1.38 M) solution were added as the initiator. Polymerization was carried on in the nitrogen environment for 24 h at 0 °C. After reaction, a transparent jelly-like product was obtained. The product was placed in deionized water and processed by an ultrasonic oscillator to remove the unreacted monomers. The product was purified by repeatedly replacing deionized water several times in the previous processing. Finally, the product was dried in an oven at 65 °C for 24 h. Poly(CD-NIPAM) and poly(CD-NIPAM-IAM) hydrogels were obtained named as samples CDg-0, CDg-1.7, CDg-2.5 and CDg-5 for allyloxy-CD/NIPAM/IAM = 1/100/0, 1/100/1.7, 1/100/2.5 and 1/100/5, respectively.

### 2.5. Identification and Characterization

Nuclear magnetic resonance (NMR) spectra were measured using a Bruker Advance 300 MHz NMR spectrometer (Bruker, Billerica, MA, USA) by weighing 10 mg of a test sample and dissolving in 1 mL of DMSO-d_6_ placed in a standard 507-HP NMR test tube.

FT-IR spectra were measured using a Perkin Elmer Spectrum RXI Fourier transform infrared (FTIR, Perkin Elmer, Waltham, MA, USA) spectrometer within 4000–400 cm^−1^ having resolution of 4.00 cm^−1^. The sample was measured using attenuated total reflection (ATR).

The weight average molecular weight (*M*_w_), number average molecular weight (*M*_n_) and PDI (*M*_w_/*M*_n_) of the polymers were determined by a Viscotek GPCmax VE-2001 (Gel permeation chromatography) system from Malvern Ltd. Co. (Malvern, UK) using polystyrene as the standard. The test sample was prepared by weighing 25 mg of a test material and dissolving in 5 mL of DMF under the conditions that a column 8 mm × 300 mm (diameter × length) and a flow rate of 1 mL/min were used, the temperature of the column was set to 50 °C, the temperature of the detector was set to 50 °C, and the injection quantity of the test sample and the standard, separately, was 50 μL.

Each of the LCSTs was determined from the transmittance of the test sample containing poly(CD-NIPAM) and poly (CD-NIPAM-IAM) copolymers as a function of temperature using a laser transmittance meter (LASOS LGK 7628), that is, the LCST is determined as the temperature when the transmittance of the solution is 50%. The test samples containing poly(CD-NIPAM) and poly(CD-NIPAM-IAM) copolymers with different concentration (1 wt %, 3 wt %, 5 wt %) were prepared by adding poly(CD-NIPAM) or poly (CD-NIPAM-IAM) copolymers (CD-0, CD-8, CD-10 and CD-12) to water in a 1 mL vial. Besides, the samples containing poly(CD-NIPAM) or poly (CD-NIPAM-IAM) copolymers (CD-0, CD-8, CD-10 and CD-12) in the solutions having different pH values (4, 7 and 10) were prepared as a 3 wt % polymer solution in the buffer solutions (pH = 4, 7 and 10).

Transmission electron microscopic (TEM) images of copolymers were observed using a high resolution transmission electron microscope (Hitachi H-7100, Hitachi, Ltd., Tokyo, Japan). The copolymer was prepared as a 1 wt % aqueous solution and 10 μL of the solution was drawn by a micro-syringe and dropped on a carbon coated copper mesh. The mesh was left standing still for 5 min and the residual liquid thereon was soaked by a filter paper. The test sample on the mesh was ready for TEM observation.

Scanning electron microscopic (SEM) images of copolymers were captured by a Hitachi S-4800 Field Emission Scanning Electron microscope (Hitachi, Ltd.). The sample to be observed was mounted on the observation stage with a double-sided carbon tape and coated gold.

Swelling ratios of hydrogels were determined by weighing a dried gel sample, dissolving the dried gel sample in deionized water, removing the swelled gel after 24 h from deionized water, eliminating water on the surface thereof and measuring the weight difference between the swelled gel and the dried gel sample to calculate the swelling ratio according to the following Equation (1).

Swelling ratio (%) = (weight of the swelled gel − weight of the dried gel)/weight of the dried gel × 100%(1)

The swelling ratios in buffer solutions (pH = 4, 7 and 10) were determined by dissolving the dried gel samples in 5 mL of buffer solutions (pH = 4, 7 and 10), respectively, removing the swelled gel samples after 24 h from the buffer solutions, eliminating the solution on the surface thereof and measuring the weight difference according to the above equation.

The swelling rates of the above hydrogel samples were measured by weighing the swelled gel samples at 10, 20, 30, 40, 60, 120, 240, 360, 480, 600, and 1440 min after being placed in the buffer solutions and wiping out the solution thereon. The results were obtained by averaging 3 time measurements.

### 2.6. Drug Release

#### 2.6.1. Encapsulation

Atorvastatin, primarily used as a lipid-lowering drug, was used as a representative drug to be carried by the newly synthesized hydrogels in this work. Atorvastatin was added to each hydrogel to have a weight ratio of atorvastatin to hydrogel be 30 wt %. Two milliliters of deionized water was added to the mixture. The resulting mixture was stirred for 48 h and the residual solution was filtered and removed. The sample was dried in a freeze-dryer to finally obtain a cotton-like drug carrier (weighed as *W*_encap_) for the drug release experiment. The drug carrier was dissolved in a buffer solution for 1 day and the upper layer was used to evaluate the encapsulation efficiency and calculate the amount of the drug which was encapsulated by the hydrogel. The encapsulation efficiency (EE) is defined as follows:

(2) $$\mathrm{EE}\left(\%\right)\;=\;\frac{W_{\mathrm{encap}}}{W_{\mathrm{drug}}}\times 100\%$$

where EE represents encapsulation efficiency, *W*_encap_ represents weight after encapsulation and *W*_drug_ represents initial weight of the drug. Three time measurements were carried out for each case.

#### 2.6.2. Drug Release Experiment

Atorvastatin has a maximum absorbance peak at 240 nm in the ultraviolet-visible (UV-Vis) spectrum and thus a UV absorbance vs. drug concentration calibration curve for atorvastatin was made. Specifically: (1) Solutions with various concentration of atorvastatin were prepared by weighing atorvastatin to dissolve in water; (2) the absorbance of the solutions was measured using a UV-Vis spectrometer at 240 nm; and (3) the calibration curve of atorvastatin in water was obtained by linear regression with an *R*^2^ value of 0.997. Similarly, the previously prepared solutions with various concentrations of atorvastatin were used and dissolved in the buffer solution pH = 2 or pH = 7.4; the absorbance of the solutions was measured; and the calibration of atorvastatin in the buffer solution pH = 2 or pH = 7.4 was obtained by linear regression with an *R*^2^ value of 0.996 or 0.997, respectively.

1 mg of the dried drug carrier was dispersed in 10 mL of buffer solutions pH 2 and pH 7.4 (PBS; phosphate buffered saline) in a flask and then the resulting mixture was placed in a water bath kept at a constant temperature of 37 °C and ultrasonic oscillation. The drug was released through diffusion. At fixed intervals, 1 mL of liquid in the flask was taken to measure the concentration of atorvastatin by the UV-Vis spectrometer based on the previous calibration curves and 1 mL of the same buffer solution was added into the flask at the same time. A cumulative release (CR) rate can be calculated as follows:

(3) $$\mathrm{CR}\left(\%\right)\;=\;\frac{W_{\mathrm{release},n}}{W_{\mathrm{encap}}}\times 100\%$$

where *W*_release,*n*_ = *V*_s_ × (*C*_1_ +…+ *C_n_*_-1_) + *V* × *C_n_*; *W*_release,*n*_ (mg) is the weight of atorvastatin taken from the sample for the *n*th time; *C_n_* (mg/mL) is the concentration of atorvastatin taken from the sample for the *n*th time; *V*_s_ (mL) is the volume taken from the sample each time; and *V* (mL) is the total volume of sample.

## 3. Results and Discussion

### 3.1. NMR and FTIR Results of Modified β-Cyclodextrin (β-CD)

**Figure 2:**
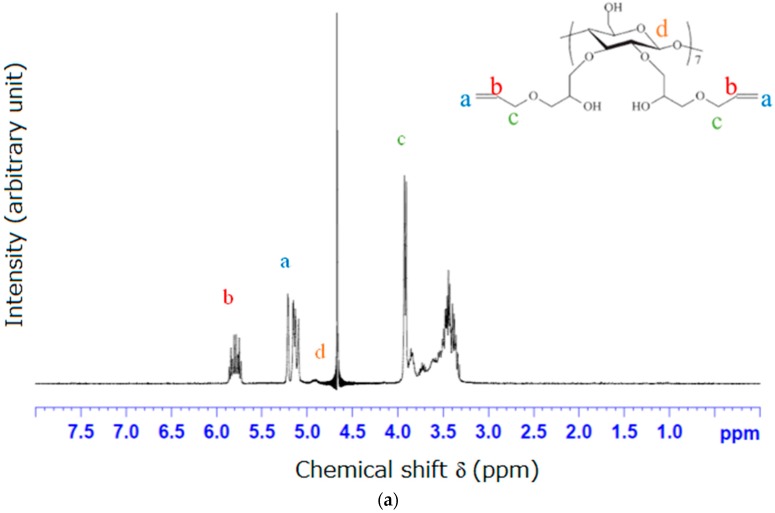

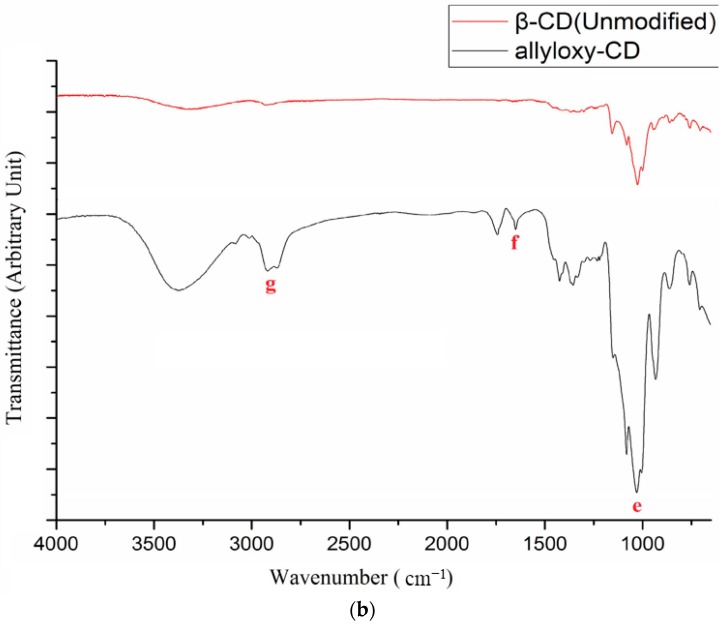
(**a**) ^1^H-nuclear magnetic resonance (^1^H-NMR) spectrum of allyloxy-CD; and (**b**) Fourier transform infrared (FTIR) spectra of unmodified β-CD and allyloxy-CD.

Figure 2a shows the ^1^H-NMR spectrum of allyloxy-CD and Figure 2b shows the FTIR spectrum of allyloxy-CD. The allyloxy-CD is the product of the reaction of β-CD with allyl glycidyl ether (AGE). The characteristic peak at δ = 2.49 ppm is attributed to the solvent, DMSO-d_6_, and the peak at δ = 3.4 ppm is attributed to H_2_O. The assignments of ^1^H-NMR spectrum of allyloxy-CD are also shown in Figure 2a. As shown in Figure 2a, the characteristic peaks at δ = 5.1–5.3 ppm (band “a”) and δ = 5.8–6.0 ppm (band “b”) are attributed to double bonds (–CH=CH_2_), the band “c” (δ ~ 4.0 ppm) is attributed to CH_2_ next to double bonds, the band “d” (δ ~ 5.0 ppm) is attributed to hydrogen on C_1_ carbon of β-CD and the peaks at δ = 3.0–3.7 ppm are attributed to the unreacted hydrogen of β-CD and new hydrogen after modification. According to the peak integration analysis, there are fourteen modified hydroxyl groups, and twelve of them are converted to double bonds.

As shown in Figure 2b, the C–O–C stretching peak is shown at 1003 cm^−1^ (band “e”) which is broad, the C=C stretching peak is shown at 1645 cm^−1^ (band “f”), and the C–H stretching peak is shown at 2800–2900 cm^−1^ (band “g”), indicating successful modification of β-CD.

### 3.2. NMR and FTIR Results of Poly(CD-NIPAM) and Poly(CD-NIPAM-IAM) Copolymers (CD-0, CD-8, CD-10 and CD-12)

**Figure 3:**
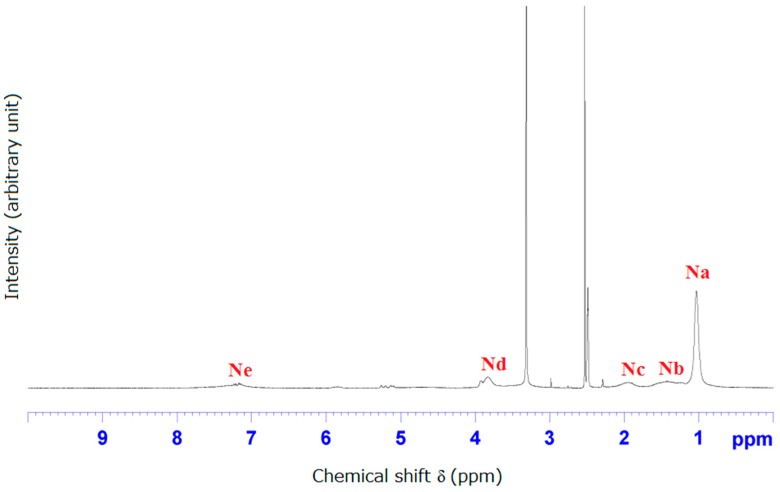
^1^H-NMR spectrum of poly(CD-NIPAM).

Figure 3 shows ^1^H-NMR spectrum of poly(CD-NIPAM) (CD-0). As shown in Figure 3, the peak at δ = 0.8–1.2 ppm (band “Na”) is attributed to –(CH_3_)_2_, the peak at δ = 1.2–1.8 ppm (band “Nb”) is attributed to –CH_2_CH–, the peak at δ = 1.8–2.2 (band “Nc”) ppm is attributed to –CH_2_CH–, the peak at δ = 3.6–4.0 ppm (band “Nd”) is attributed to –CH(CH_3_)_2_, and the peak δ = 7.0–8.0 ppm (band “Ne”) is attributed to –CONH from NIPAM.

**Figure 4:**
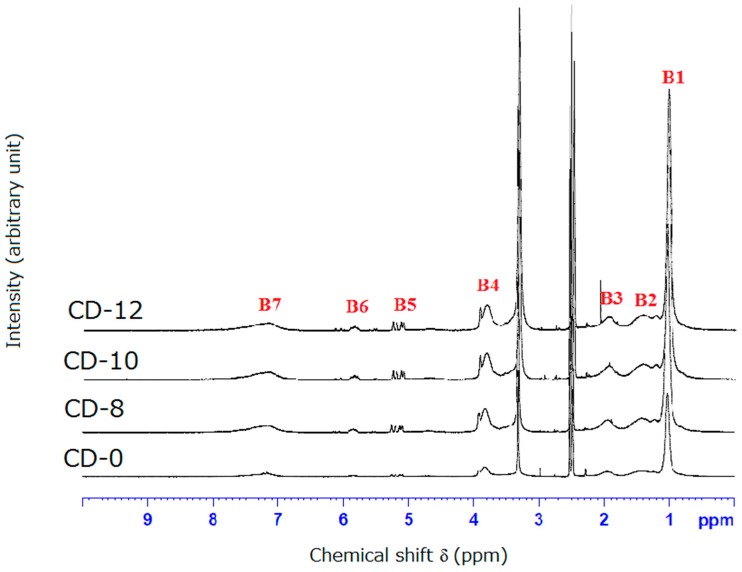
^1^H-NMR spectra of poly(CD-NIPAM-IAM) copolymers.

Figure 4 shows ^1^H-NMR spectra of poly(CD-NIPAM-IAM) copolymers (CD-8, CD-10 and CD-12). As shown in Figure 4, the characteristic peaks from IAM are at δ = 1.2–1.8 ppm (–CH_2_CH) (band “B2”), δ = 1.8–2.2 ppm (–CH_2_–CO–NH_2_) (band “B3”) and δ = 7.0–8.0 ppm (–CH_2_–CO–NH_2_) (band “B7”) and the characteristic peaks from NIPAM are at δ = 0.8–1.2 ppm (band “B1”) is attributed to –NH–CH–(CH_3_)_2_, the peak at δ = 1.2–1.8 ppm (band “B2”) is attributed to –CH_2_CH–CO–, the peak at δ = 1.8–2.2 ppm (band “B3”) is attributed to –CH_2_CH–CO, the peak at δ = 3.6–4.0 ppm (band “B4”) is attributed to –NH–CH(CH_3_)_2_, and the peak δ = 7.0–8.0 ppm (band “B7”) is attributed to –NH–CH–(CH_3_)_2_. The characteristic peaks (bands “B2”, “B3” and “B7”) from IAM and NIPAM in poly(CD-NIPAM-IAM) copolymers are overlapping. The characteristic peaks attributed to IAM and NIPAM composition units are consistent with the previous studies [23,24].

The bands at δ = 5.0–5.3 ppm (band “B5”) and δ = 5.7–5.9 ppm (band “B6”), attributed to –CH_2_=CH–CO– and –CH_2_=CH–CO–, respectively, was shown in ^1^H-NMR spectrum of CD-0 as well as CD-8, CD-10 and CD-12, indicating the double bonds of allyloxy-CD were not completely reacted. However, the number of residual double bonds is not enough to be used to produce cross-linked hydrogels.

The ratio of the composition units from NIPAM to the composition units from IAM in CD-8, CD-10 and CD-12 was determined by ^1^H-NMR where the area of the peak at δ = 3.6–4.0 ppm (–CH–NH–) is defined as 1 and the peaks at δ = 1.2–1.8 ppm (2H from NIPAM and 2H from IAM) and δ = 1.8–2.2 ppm (1H from NIPAM and 2H from IAM) are used to quantize the composition units from NIPAM and the composition units from IAM. That is, if H*x* represents H from NIPAM and H*y* represents H from IAM, the following two equations will be obtained: 2H*x* + 2H*y* = ING1 and H*x* + 2H*y* = ING2 where ING1 and ING2 are the value from integrating the area of the peak at δ = 1.2–1.8 ppm and δ = 1.8–2.2 ppm, respectively. However, since the peaks at δ = 1.2–1.8 ppm and δ = 0.8–1.2 ppm are highly overlapping and the peaks δ = 1.2–1.8 ppm and δ = 1.8–2.2 ppm are also overlapping, the calculated polymerized ratio of NIPAM/IAM is considered unreliable.

### 3.3. FTIR Results of Poly(CD-NIPAM) and Poly(CD-NIPAM-IAM) Copolymers

**Figure 5:**
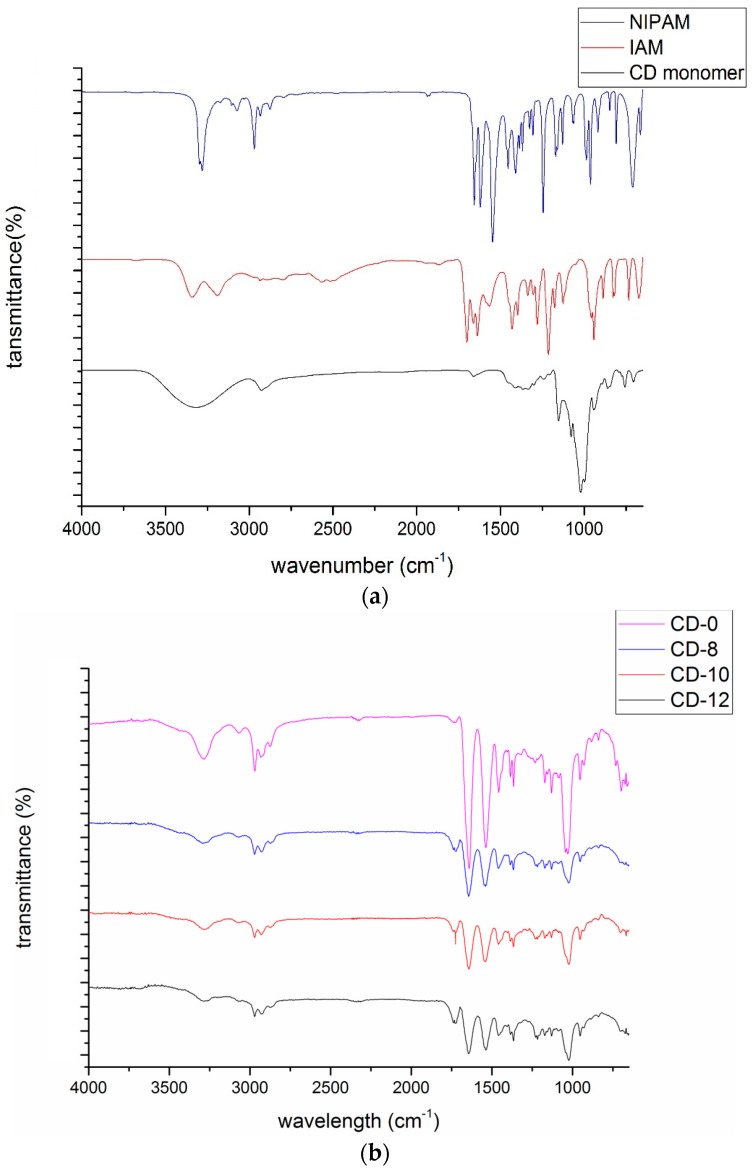

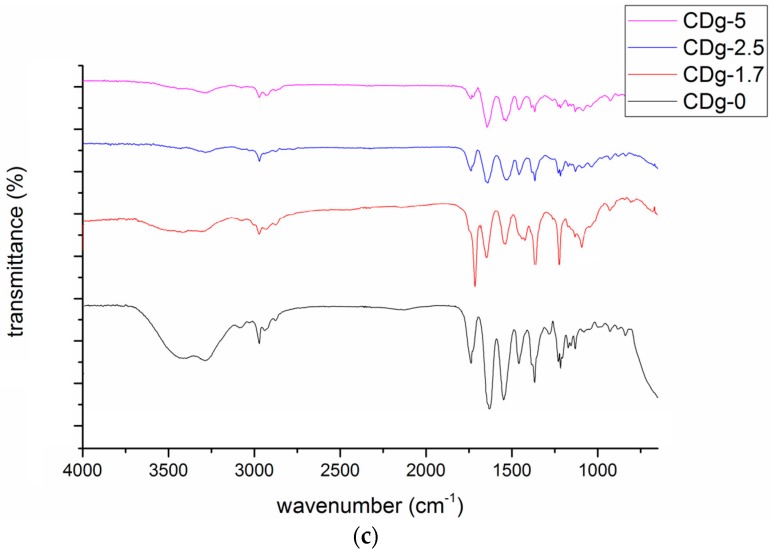
FTIR Spectra of: (**a**) NIPAM, IAM and unmodified β-CD monomers; (**b**) poly(CD-NIPAM-IAM) copolymers; and (**c**) poly(CD-NIPAM-IAM) hydrogels.

The FTIR spectra of poly(CD-NIPAM) and poly(CD-NIPAM-IAM) copolymers, CD-0, CD-8, CD-10 and CD-12, are shown in Figure 5b. The FTIR spectra of the NIPAM, IAM and unmodified β-CD monomers are shown in Figure 5a. The FTIR spectrum of the modified β-CD is shown in Figure 1c.

In the spectrum of NIPAM, the amide C=O stretching peak is shown at 1654 cm^−1^, the amine N–H bending peak is shown at 1549 cm^−1^, the amine N–H stretching peak is shown at 3280 cm^−1^ and the mono-substituted C=C (vinyl) peak is shown at 990 cm^−1^. In the spectrum of IAM, the C=O stretching peak is shown at 1710 cm^−1^ and the O–H stretching peak is shown at 3300 cm^−1^. In the spectrum of unmodified β-CD, the broad O–H stretching peak is shown at 3300 cm^−1^. After modification, as shown in the spectrum of allyloxy-CD, the C=C stretching peak is shown at 1645 cm^−1^, indicating successful modification of β-CD. After polymerization, the spectra of poly(CD-NIPAM) and poly(CD-NIPAM-IAM) copolymers show an absence of the 990 cm^−1^ peak indicating C=C is formed into C–C.

As shown in Figure 5b, the spectra of the copolymers show the intensity of the 1710 cm^−1^ peak (due to C=O stretch of carboxylic group of IAM) increases with the molar fraction of IAM but the quantitative determination of the IAM molar fraction cannot be achieved from the FTIR spectra, particularly by ATR-FTIR spectra. Since the N-H stretching peak and the O-H stretching peak are overlapped at about 3300 cm^−1^, the increasing trend attributing to the increase of the IAM molar fraction is not clearly shown. In addition to the above NMR spectra, the FTIR spectra also confirm successful synthesis of poly(CD-NIPAM) and poly(CD-NIPAM-IAM) copolymers.

Figure 5c shows the FTIR spectra of poly(CD-NIPAM-IAM) hydrogels, CDg-0, CDg-1.7, CDg-2.5 and CDg-5. As shown in Figure 5c, the intensity of the 1710 cm^−1^ peak (due to C=O stretch of carboxylic group of IAM) also shows an increasing trend with the increase of the IAM molar fraction.

### 3.4. GPC Analysis

The weight average molecular weight (*M*_w_), the number average molecular weight (*M*_n_) and PDI (*M*_w_/*M*_n_) calculated from GPC results are shown in Table 1. As the molar fraction of IAM increases, the molecular weight of the copolymer is higher which may be because of hydrogen bond formation between the solvent (DMSO) and the copolymer which has more hydrophilic groups derived from IAM or more proton-donor groups (NH_2_ and COOH). The two-component copolymer, poly(CD-NIPAM), has smaller PDI (about 1.54) than the tri-component copolymer, poly(CD-NIPAM-IAM). For samples CD-8, CD-10 and CD-12, PDI is always about 2.0 regardless of the molar fraction of IAM. The molecular weight of the copolymer increases with the increase of the molar fraction of IAM, indicating IAM co-monomer has the higher reactivity than NIPAM which is also found true in the previous studies [23,24].

### 3.5. LCST Analysis

**Figure 6:**
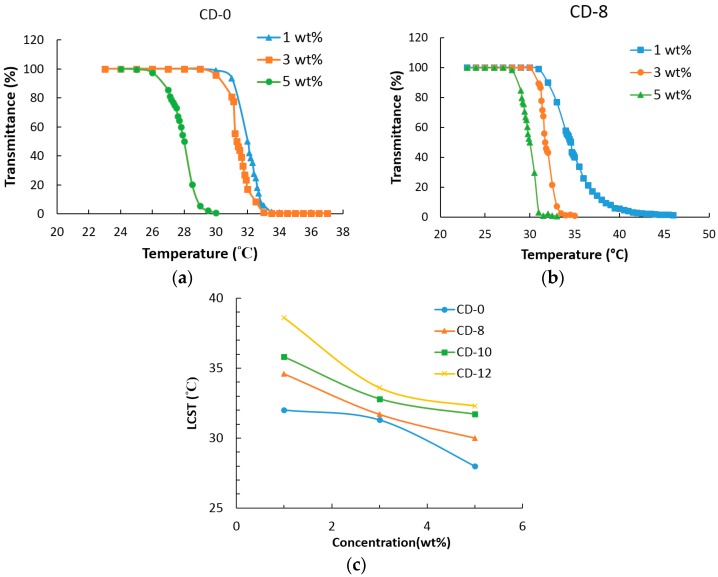
Plots of: (**a**) transmittance vs. temperature of sample CD-0; (**b**) transmittance vs. temperature of sample CD-8; and (**c**) lower critical solution temperatures (LCST) vs. concentration for poly(CD-NIPAM) and poly(CD-NIPAM-IAM) copolymers.

Figure 6a,b shows the plots of transmittance vs. temperature of samples CD-0 and CD-8. The LCST is determined to be the temperature when the transmittance of the solution is 50%. The LCSTs of the 1 wt %, 3 wt % and 5 wt % copolymer solutions are shown in Figure 6c and Table 2. Figure 6c shows the plots of LCST vs. concentration for poly(CD-NIPAM) and poly(CD-NIPAM-IAM) copolymers, CD-0, CD-8, CD-10 and CD-12. The effect of concentration on LCST for various copolymers, samples CD-0–CD-12, is similar; that is, the largest difference on LCST (ΔLCST) shown in Table 2 for samples CD-0–CD-12 is relatively close to each other.

The LCST decreases with the increase of the concentration for sample CD-0, which may be because the molecular chains are closer to each other at the higher concentration to cause the hydrophobic moieties of NIPAM composition units to easily get more entangled and gather together so that the interaction force between the molecules is larger than the strength of the hydrogen bonds with water to expel water molecules from the copolymer [23]. For sample CD-8, as shown in Figure 6b and Table 2, the LCST has a similar trend as that of sample CD-0. As shown in Table 2 and Table 3, compared to CD-0 containing no IAM, sample CD-8 has the higher LCST.

As shown in Figure 6c, the LCST decreases with the increase of the concentration for samples CD-0, CD-8, CD-10 and CD-12. Besides, at the same concentration, the LCST has an increasing trend with the addition amount of IAM (IAM molar fraction). Therefore, the above result indicates that the concentration of the solution and the molar fraction of IAM are two factors to adjust the LCSTs.

**Figure 7:**
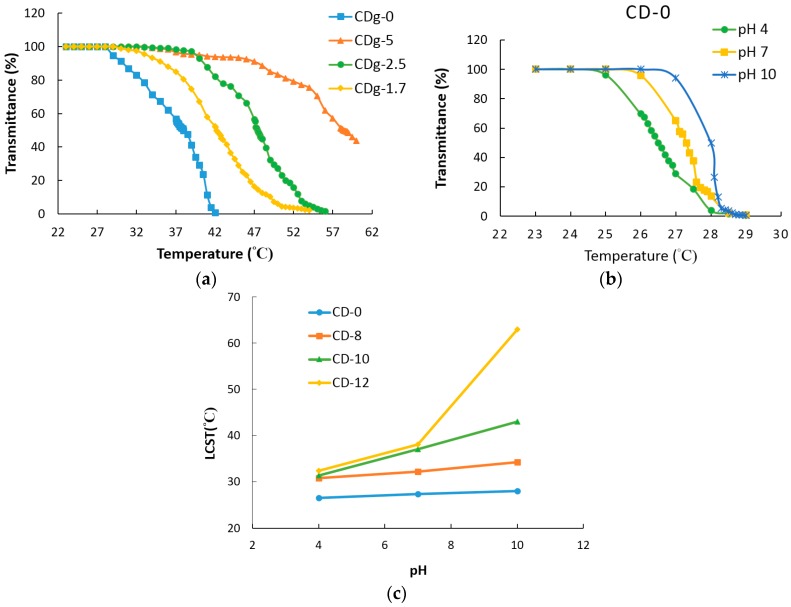
Plots of: (**a**) transmittance vs. temperature of poly(CD-NIPAM) and poly(CD-NIPAM-IAM) hydrogels; (**b**) transmittance vs. temperature of sample CD-0 in various buffer solutions (pH = 4, 7 and 10); and (**c**) LCST vs. pH value of 3 wt % poly(CD-NIPAM) and poly(CD-NIPAM-IAM) copolymers in various buffer solutions.

Figure 7a shows the plots of transmittance vs. temperature of poly(CD-NIPAM) and poly(CD-NIPAM-IAM) hydrogels, CDg-0, CDg-1.7, CDg-2.5 and CDg-5. The hydrogels were formed directly in the test vials for phase transition measurements. The vials were wrapped in a heating bag to adjust the environmental temperature. The plots of transmittance vs. temperature of the hydrogels show thermal sensitive hydrogels and the swelling property of the hydrogel is varied with the change of temperature to result in water absorption and elimination. Since the hydrogels have cross-linking structures because of addition of the cross-linking agent during copolymerization, the phase transition temperature are much higher than the linear copolymers thereof. At the low temperature, the hydrogel appears transparent and colorless because the amide bonds of NIPAM composition units form hydrogen bonds with water. On the other hand, at the high temperature, the interaction of hydrophobic ends of NIPAM composition units is larger than the hydrogen bonding with water to make the hydrogels become opaque creamy white. As shown in Figure 7a, the LCSTs of hydrogels CDg-1.7, CDg-2.5 and CDg-5 are much larger than that of CDg-0. For hydrogels CDg-1.7, CDg-2.5 and CDg-5, the LCST has an increasing trend with the increase of the molar fraction of IAM. Table 3 shows the LCSTs and the feed NIPAM/IAM ratios of CDg-0, CDg-1.7, CDg-2.5 and CDg-5.

The pH dependence of LCST was investigated. Figure 7b shows the plots of transmittance vs. temperature of 3 wt % sample CD-0 in various buffer solutions (pH = 4, 7 and 10). The LCSTs of 3 wt % copolymers in various buffer solutions (pH = 4, 7 and 10) and the LCST difference are shown in Table 4. The difference of LCSTs of poly(CD-NIPAM) copolymer (CD-0) either in the acidic or alkaline environment is only about 2 °C, since CD-0 has no significant dependence on pH values and is not pH sensitive due to no IAM composition unit and the LCST of CD-0 is stable either in the acidic or alkaline environment.

Figure 7c shows the plots of LCST vs. pH value of 3 wt % poly(CD-NIPAM) and poly(CD-NIPAM-IAM) copolymers in various buffer solutions. It is found from Table 4 that the more the addition quantity of IAM, the more the difference in LCST, which may be because the carboxylic group of IAM is affected by the pH value of the solution to be protonated or deprotonated to consequently affect the phase transition temperature. That is, for example, at a low pH value, less carboxylic groups from IAM composition units in the copolymer are charged and lead to enhance the formation of hydrogen bonds between COOH moiety from IAM and NHCO moiety from NIPAM to make the copolymer have lower water solubility so that the LCST is lowered. Thus, the LCST is higher at a high pH value and the trend is more significant for the copolymer having a higher molar ratio of IAM/NIPAM. Zhao et al. [26] reported that the phase transition behavior of a thermo- and pH-sensitive NIPAM-based microgel containing poly(l-glutamic acid) showed the increasing trend of LCST with the increased pH value. However, in the current study, the content of IAM in the copolymer necessary to show pH sensitivity of the copolymer is much less than using poly(l-glutamic acid) that reported by Zhao et al. [26].

### 3.6. Morphological Analysis

**Figure 8:**
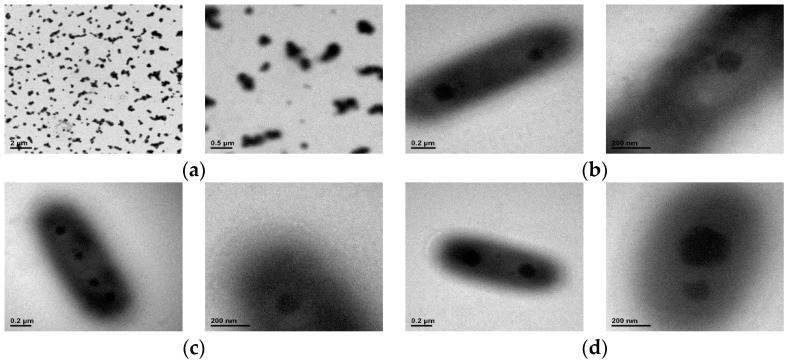
Transmission electron microscopic (TEM) images of: (**a**) CD-0; (**b**) CD-8; (**c**) CD-10; and (**d**) CD-12.

Figure 8 shows TEM images of (Figure 8a) CD-0, (Figure 8b) CD-8, (Figure 8c) CD-10, and (Figure 8d) CD-12 with two different magnification ratios (Figure 8, left: 30,000× and right: 60,000×). Since the sample was observed under a vacuum and dried environment, the particle of the copolymer was dried and the dehydration between molecular chains was expected so that a smaller particle size is shown. A polymer could form into micelles driven by the hydrophobic effect and self-assembly to form various types of nanostructures, such as spherical or worm-like micelles or vesicles [30] depending on the composition and actual self-assembly of the polymer. As shown in Figure 8a, since no hydrophilic IAM was contained in sample CD-0, the TEM image of CD-0 does not show a core–shell structure. For the addition of IAM, as shown in Figure 8b–d, the TEM images show the micelle is encircled by a light-colored rim, indicating the hydrophilic IAM affects the self-assembly behavior of the molecular chain and is driven to face outside and the hydrophobic NIPAM stays inside. Besides, as the molar fraction of IAM increases, the TEM images show the light-colored rim becomes more obvious.

**Figure 9:**
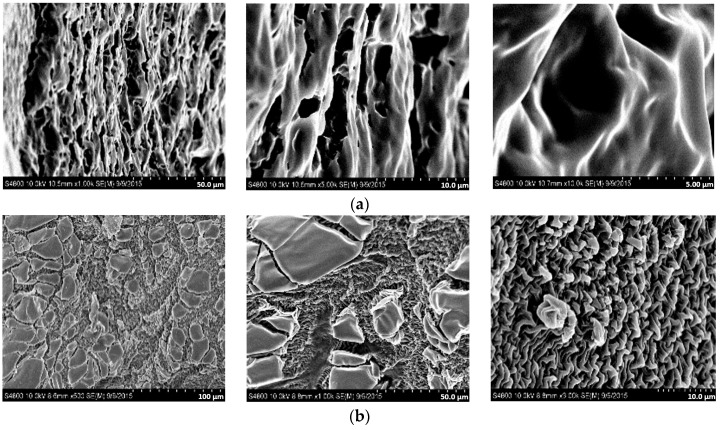

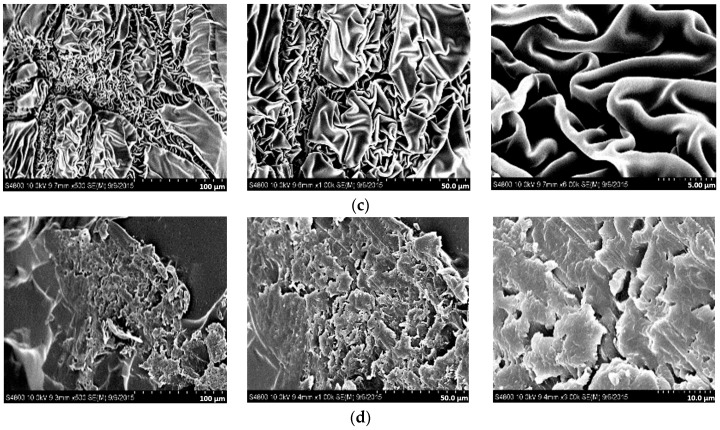
Scanning electron microscopic (SEM) images of: (**a**) CDg-0; (**b**) CDg-1.7; (**c**) CDg-2.5; and (**d**) CDg-5.

The morphology of the hydrogels was observed by SEM. Since the hydrogels were copolymerized together with a cross-linking agent, the molecular network structures were investigated by SEM. Figure 9 shows SEM images of (Figure 9a) CDg-0, (Figure 9b) CDg-1.7, (Figure 9c) CDg-2.5, and (Figure 9d) CDg-5 with three different magnification ratios (Figure 9, left: 500×, middle: 1000× and right: 6000×). In Figure 9a, the SEM images of sample CD-0 shows small pores since there is no hydrophilic IAM. As the addition quantity of IAM increases, the pores become the sheet-like structures, especially for CDg-5 which has the highest IAM molar fraction. Such a sheet-like structure may enhance the swelling property of the hydrogel.

### 3.7. Swelling Property of Hydrogels

**Figure 10:**
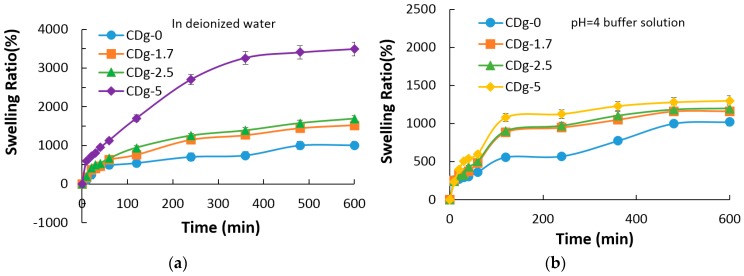

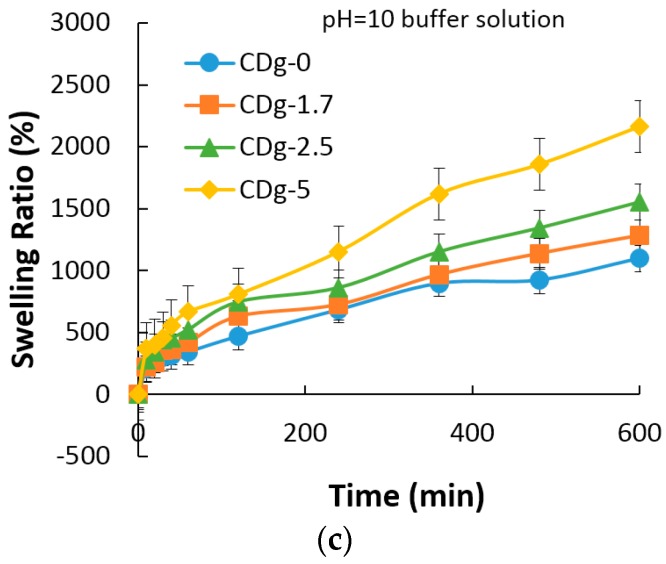
Swelling ratio vs. time curves for hydrogels: (**a**) in deionized water; (**b**) in the pH 4 buffer solution; and (**c**) in the pH 10 buffer solution with a time span up to 600 min.

Figure 10 shows swelling ratio vs. time curves for hydrogels CDg-0, CDg-1.7, CDg-2.5 and CDg-5 (Figure 10a) in deionized water, (Figure 10b) in the pH 4 buffer solution, and (Figure 10c) in the pH 10 buffer solution with a time span up to 600 min. The swelling ratio of the hydrogel increases with the increase of the molar fraction of IAM. Specifically, the swelling ratio of CDg-0 containing no IAM is up to 1009% and that of CDg-5 having the highest molar fraction of IAM reaches about 3500%. Regarding the swelling ratio, as shown in Figure 10, the swelling rate of hydrogel CDg-5 is the highest initially while the swelling rates of the other hydrogels are close to each other and almost have no distinct difference.

**Figure 11:**
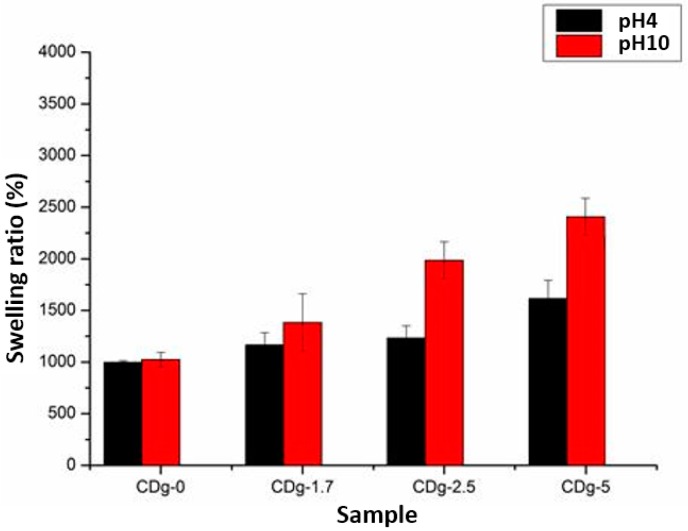
Swelling ratios of hydrogels in buffer solution (pH = 4 and 10).

In addition to the swelling ratio in deionized water, the swelling ratios of hydrogels in acidic and alkaline environments were investigated. Figure 11 shows the swelling ratios of hydrogels in buffer solution (pH = 4 and 10). Since the IAM composition unit in the copolymer has the carboxylic group as a COOH form in an acidic environment, the network structure of the copolymer is almost the same as the structure in deionized water. On the other hand, since the carboxylic group of IAM becomes COO^−^ form in an alkaline environment, the COO^−^ group generates repulsive force against each other to make the network structure swell to absorb more buffer solutions. Since the IAM composition unit contributes to the pH sensitivity of the copolymer, as the molar fraction of IAM increases, the copolymer has different swelling rates at different pH values. The swelling rate is more linearly depending on the molar fraction of IAM in the acidic or alkaline environment, compared to that in deionized water. Among the synthesized hydrogels, CDg-5 has the highest swelling rate as well as swelling ratio. In addition, CDg-5 shows distinct difference in swelling rates at different pH values.

### 3.8. Drug Encapsulation and Release

The calibration curves of atorvastatin in: (a) deionized water; (b) pH 2 buffer solution; and (c) pH 7.4 buffer solution, measured by a SHIMADZU UV-3600 UV-Vis spectrophotometer for the subsequent drug release investigation, are provided in the Supplementary Materials (Figure S1). The calibration curves of atorvastatin at pH = 2 and 7.4, simulating the environment of the human gastric fluid or the intestinal fluid were used to calculate the release rate of atorvastatin in the acidic (gastric fluid) or neutral (intestinal fluid) environments.

The encapsulation efficiency (%) of various initial amount of atorvastatin for sample CDg-0 is provided in the Supplementary Materials (Figure S2). The encapsulation efficiency (%) is above 90%, which may be because atorvastatin has a large amount of hydrogen bonding acceptors to bond with β-CD. It was found that the encapsulation efficiency (%) is the highest being about 95.6% when the drug concentration reaches 30 wt %. However, as the concentration is higher than 30 wt %, the encapsulation efficiency (%) decreases, indicating the limit of the drug amount the carrier can carry. Therefore, the concentration of atorvastatin is chosen to be 30 wt % for the subsequent drug release experiment.

The encapsulation efficiency (%) of atorvastatin for various hydrogel carriers is shown in the Supplementary Materials (Figure S3). As the molar fraction of IAM increases, the encapsulation efficiency decreases. The hydrogel CDg-5 shows the smallest encapsulation efficiency among the synthesized hydrogels. However, the difference in the encapsulation efficiency is not significant and the encapsulation efficiency of CDg-5 is still about 94% which is about 2% less than CDg-0.

**Figure 12:**
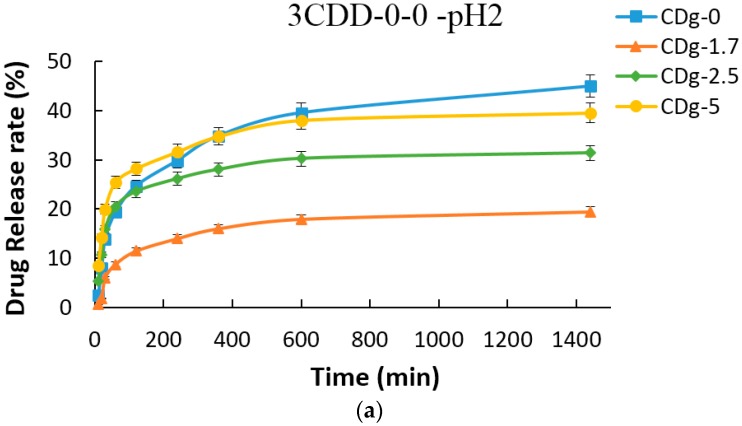

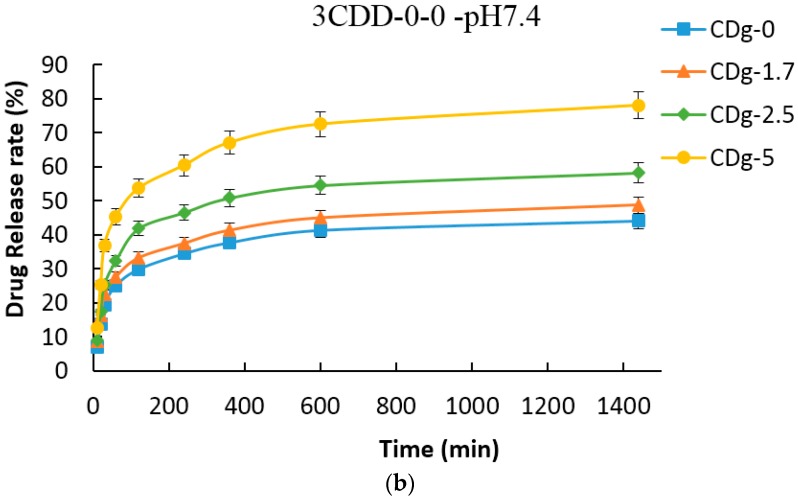
Drug release rates of various hydrogel carriers at 37 °C and: (**a**) pH = 2; and (**b**) pH = 7.4.

In the drug release experiment, 37 °C was chosen to simulate the human body temperature and the pH = 2 and pH = 7.4 buffer solutions were used to simulate acidic (gastric fluid) or neutral (intestinal fluid) environments, respectively. Figure 12 shows the drug release rates of various hydrogel carriers at 37 °C and (Figure 12a) pH = 2 and (Figure 12b) pH = 7.4. In the pH = 2 environment, CDg-0 has the quickest release rate and releases 45.0% of drug after 1440 min while CDg-1.7 has the slowest release rate and releases 19.5% of drug after 1440 min. After 1440 min, CDg-2.5 and CDg-5 release 31.4% and 39.6% of drug, respectively. In the pH = 7.4 environment, CDg-5 has the quickest release rate and releases 78.1% of drug after 1440 min while CDg-0 has the slowest release rate and releases 44.1% of drug after 1440 min. After 1440 min, CDg-1.7 and CDg-2.5 release 48.8% and 58.3% of drug, respectively. CDg-0 has no significant difference in drug release rates between the pH = 2 and pH = 7.4 environments because CDg-0 contains no IAM composition unit which causes the copolymer to be pH sensitive or responsive. On the contrary, the hydrogels containing IAM have distinct difference in drug release rates in the environments of different pH values. Specifically, CDg-5 has the quickest release rate in the pH = 7.4 environment. Therefore, the copolymers synthesized in this work are suitable to be used as drug carriers to successfully resist the acidic environment to keep the encapsulated drug and release it in the neutral or alkaline environment. 

## 4. Conclusions

In the current work, thermo- and pH-sensitive copolymers and hydrogels poly(β-CD-NIPAM-IAM) were prepared by radical polymerization. β-CD monomer was modified before radical polymerization. The composition unit from β-CD monomer was incorporated in order to aid the drug encapsulation applicability of the copolymer. The hydrogels were copolymerized together with a cross-linking agent to have cross-linking network molecular structures. The poly(CD-NIPAM-IAM) hydrogels were first synthesized and studied in this work. The composition unit from IAM monomer contributes to the pH-sensitive property of the copolymer. The LCST of the copolymer increases as the molar fraction of IAM increases, which indicates IAM can effectively increase the LCST of the copolymer. As the concentration of the solution increases, the LCST decreases.

The TEM images of the copolymers containing IAM show the core–shell phenomenon, indicating that the addition of hydrophilic IAM enhances self-assembly of hydrophilic and hydrophobic portions of copolymers to form micelles. Although the micelle formation due to IAM composition units in the star copolymer was proposed in the previous work [24] based on the data of the particle diameter, the current work shows the synthesized copolymer has a core–shell structure. On the other hand, the random copolymer containing IAM composition units in the other previous work [23] does not show micelle formation.

As the pH value varies, the phase transition temperature of sample CD-0 has no obvious difference, indicating CD-0 has no pH sensitivity. After IAM is added, there are significant differences in LCST in buffer solutions at various different pH values.

The SEM images of the hydrogels show that the pores gradually become the sheet-like structures with the increase quantity of IAM. The sheet-like structures contribute to the increase in water absorption capability.

The different trend in LCST between the copolymers and hydrogels implies that cross-linked hydrogels have network structures to require more energy in phase transition.

The result of swelling ratios shows that the swelling rate increases as the molar fraction of IAM increases and the swelling ratio is different at different pH values because of the pH sensitivity of hydrogels. The swelling property of the hydrogel shows dependence on pH values which is useful and favorable in the application of drug delivery.

From the drug release experiment, the hydrogel carrier CDg-5 shows excellent encapsulation and has little release (less than 40%) in the simulated gastric fluid environment (pH = 2) and 78% release in the simulated intestinal fluid environment (pH = 7.4). The carrier CDg-5 is pH sensitive and can resist the acidic environment to keep the encapsulated drug and release later in the intestine. Therefore, the copolymers synthesized in this work are useful as a drug carrier to release in the intestine.

## Figures and Tables

**Table 1:** Molecular weight determination.

Sample	*M*_w_	*M*_n_	PDI (*M*_w_/*M*_n_)
CD-0	59,500	38,600	1.54
CD-8	123,000	62,900	1.96
CD-10	162,600	79,000	2.06
CD-12	167,500	84,700	1.98

**Table 2:** Lower critical solution temperatures (LCSTs) of 1 wt %, 3 wt % and 5 wt % copolymer solutions.

Sample	1 wt %	3 wt %	5 wt %	ΔLCST
CD-0	32.0 °C	31.3 °C	28.0 °C	4 °C
CD-8	34.6 °C	31.7 °C	30.0 °C	4.6 °C
CD-10	35.8 °C	32.8 °C	31.7 °C	4.1 °C
CD-12	38.6 °C	33.6 °C	32.3 °C	6.3 °C

**Table 3:** NIPAM/IAM ratios and LCSTs of hydrogels.

Sample	NIPAM/IAM	LCST
CDg-0	100/0	38 °C
CDg-1.7	100/1.67	42.4 °C
CDg-2.5	100/2.5	47.4 °C
CDg-5	100/5	58.3 °C

**Table 4:** LCSTs of 3 wt % copolymers in various buffer solutions.

Sample	pH 4	pH 7	pH 10	ΔLCST
CD-0	26.5 °C	27.3 °C	28 °C	1.5 °C
CD-8	30.8 °C	32.3 °C	34.2 °C	3.4 °C
CD-10	31.3 °C	37 °C	43 °C	11.7 °C
CD-12	32.4 °C	38.1 °C	63 °C	30.6 °C

## Supplementary Materials

The following are available online at www.mdpi.com/1996-1944/9/12/1003/s1, Figure S1: Calibration curves of atorvastatin in: (a) deionized water; (b) pH 2 buffer solution; and (c) pH 7.4 buffer solution. Figure S2: Encapsulation efficiency (%) of various initial amount of atorvastatin for sample CDg-0. Figure S3: Encapsulation efficiency (%) of atorvastatin for various hydrogel carriers.
