# Supplementary material for: Synthesis and Drug Delivery Application of Thermo- and pH-Sensitive Hydrogels: Poly(β-CD-co-N-Isopropylacrylamide-co-IAM)
Source: Materials (Basel). 2016 Dec 11;9(12):1003. doi: 10.3390/ma9121003 (PMC5456989; doi:10.3390/ma9121003)
Supplement: Supplementary file 1 [file materials-09-01003-s001.pdf]

Supplementary Materials: Synthesis and Drug Delivery Application of Thermo- and pH-Sensitive Hydrogels: Poly(β-CD-*co*-*N*-isopropylacrylamide-*co*-IAM)

Syang-Peng Rwei, Tuan Huynh Nguyen Anh, Whe-Yi Chiang, Tun-Fun Way and Yung-Jia Hsu

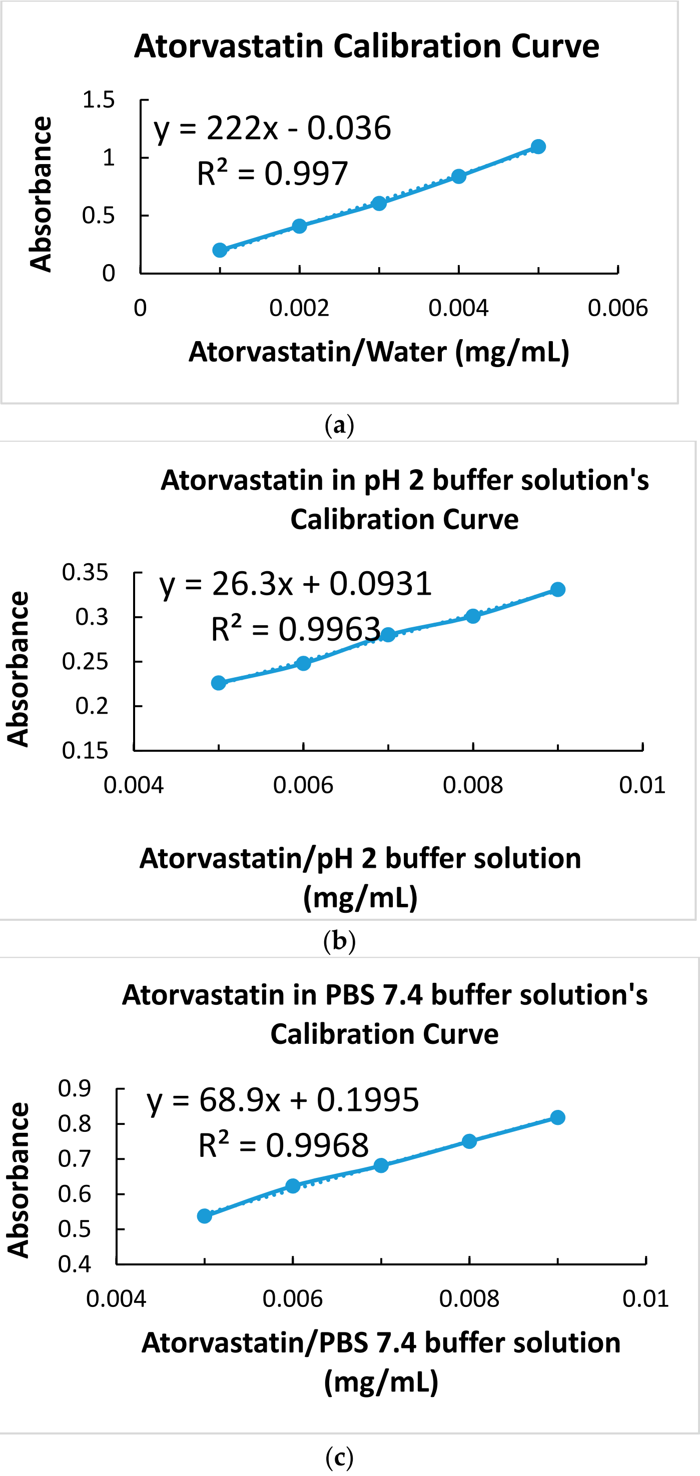

Figure S1. Calibration curves of atorvastatin in: (**a**) deionized water; (**b**) pH 2 buffer solution; and (**c**) pH 7.4 buffer solution.

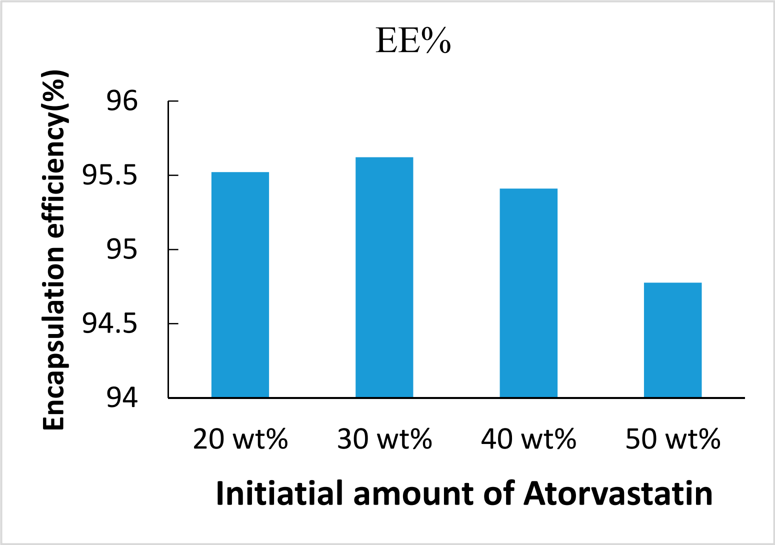

Figure S2. Encapsulation efficiency (%) of various initial amount of atorvastatin for sample CDg-0.

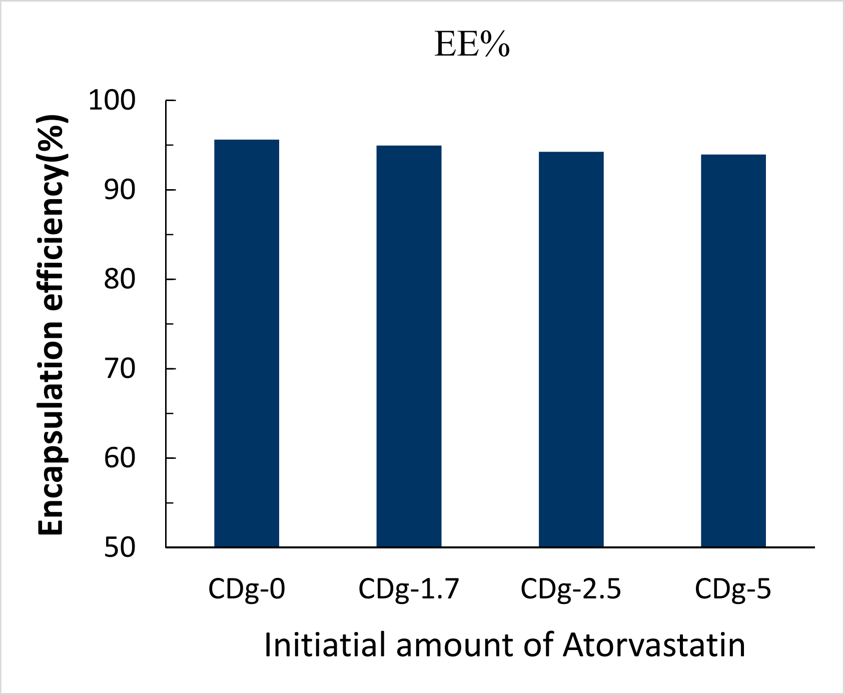

Figure S3. Encapsulation efficiency (%) of atorvastatin for various hydrogel carriers.
